# Video double-lumen tube for one lung ventilation: implementation and experience in 343 cases of routine clinical use during the first 20 months of the SARS-CoV-2 pandemic

**DOI:** 10.1186/s13019-024-02663-5

**Published:** 2024-04-16

**Authors:** Andrea Irouschek, Joachim Schmidt, Torsten Birkholz, Horia Sirbu, Andreas Moritz

**Affiliations:** 1 Department of Anesthesiology, University Hospital Erlangen, Faculty of Medicine, Friedrich-Alexander University of Erlangen-Nuremberg, Erlangen, Germany; 2 Department of Thoracic Surgery, University Hospital Erlangen, Faculty of Medicine, Friedrich-Alexander-University of Erlangen-Nuremberg, Erlangen, Germany; 3 Department of Anesthesiology, University Hospital Erlangen, Krankenhausstr. 12, 91054 Erlangen, Germany

**Keywords:** One lung ventilation, Thoracic surgery, Double-lumen tube, Video double-lumen tube, SARS-CoV-2

## Abstract

**Background:**

Double-lumen tubes (DLTs) are the preferred device for lung isolation. Conventional DLTs (cDLT) need a bronchoscopic position control. Visualisation of correct DLT positioning could be facilitated by the use of a video double-lumen tube (vDLT). During the SARS-CoV-2-pandemic, avoiding aerosol-generation was suggesting using this device. In a large retrospective series, we report both general and pandemic related experiences with the device.

**Methods:**

All anesthesia records from patients aged 18 years or older undergoing surgery from April 1st, 2020 to December 31st, 2021 in the department of thoracic surgery requiring intraoperative lung isolation were analyzed retrospectively.

**Results:**

During the investigation period 343 left-sided vDLTs (77.4%) and 100 left-sided cDLTs (22.6%) were used for one lung ventilation. In the vDLT group bronchoscopy could be reduced by 85.4% related to the cDLT group. Additional bronchoscopy to reach or maintain correct position was needed in 11% of the cases. Other bronchoscopy indications occured in 3.6% of the cases. With cDLT, in 1% bronchoscopy for other indications than conforming position was observed.

**Conclusions:**

The Ambu® VivaSight™ vDLT is an efficient, easy-to-use and safe airway device for the generation of one lung ventilation in patients undergoing thoracic surgery. The vDLT implementation was achieved easily with full interchangeability to the left-sided cDLT. Using the vDLT can reduce the need for aerosol-generating bronchoscopic interventions by 85.4%. Continuous video view to the carina enabling position monitoring of the DLT without need for bronchoscopy might be beneficial for both employee’s and patient’s safety.

## Background

Thoracic surgery often requires lung isolation to provide optimal surgical site exposure. Double-lumen tubes (DLTs) are with 95% the most common device used [1, 2]. However, its insertion and correct placement might be difficult and challenging. Visual confirmation via bronchoscopy is the widely used standard procedure [1–4].

In December 2019, the novel corona virus SARS-CoV-2 began its pandemic worldwide spread [5].This led WHO to declare a Public Health Emergency of International Concern on January 30, 2020, and to characterize the outbreak as a pandemic on March 11, 2020 [6, 7]. Beside droplet infection, aerosol transmission plays a major role. Accordingly, aerosol-generating settings in daily clinical practice were identified in order to minimize exposure. In the perioperative setting, coughing, mask bag ventilation, endotracheal intubation and especially bronchoscopic procedures were found to produce significant amounts of contagious aerosols [8–13].

The Ambu® VivaSight™ video double-lumen tube (vDLT) can avoid bronchoscopy confirming its position from a theoretical point of view. The device is available as a left-sided DLT in 35, 37, 39 and 41 French (Fr) and designed similar and interchangeably to a conventional left-sided double-lumen tube (cDLT) [14–18]. The additional feature of the vDLT consists of a small video camera at the tracheal outlet of the tracheal lumen, enabling permanent supervision of placement and the actual position of the device. Therefore, according to some preliminary experiences most bronchoscopic procedures are not required anymore [15–20]. However, large and well designed, multicenter RCT are still lacking.

Several prospective and mostly randomised studies compared vDLTs with cDLTs indicating interchangeability [15–18, 20]. Regarding daily clinical routine, only a few studies evaluated the vDLT [14, 21]. Therefore, this report should add comprehensive practical experience with implementation and use of the vDLT during the first 20 month of SARS-CoV-2 pandemic.

## Methods

After approval of the local ethics committee (reference-number: 22-25-Br), we retrospectively analyzed the electronic anesthesia records of all patients aged 18 years or older, who underwent general anesthesia with intraoperative one lung ventilation in the department of thoracic surgery of the university hospital Erlangen, a tertiary care hospital, between April 1st, 2020 to December 31st, 2021. All data were retrieved from the electronic anesthesia record data management system (NarkoData®; IMESO, Huettenberg, Germany).

Standard monitoring for general anesthesia with lung separation in thoracic surgery included electrocardiogram, pulse oximetry, capnography and invasive blood pressure (anesthesia respirator Perseus™, IACS monitoring system™, Dräger, Lübeck, Germany).

For one lung ventilation, the university hospital Erlangen provides standard operating procedure (SOP) for the use of DLT or the use of single lumen tube with a bronchial blocker. Multiple devices for lung separation (double-lumen tube: Bronchocath™, Rüsch, Teleflex Medical, Athlone, Irland; Ambu® VivaSight™ DLT, ETView Ltd., Amsterdam, The Netherlands; bronchial blocker: Arndt-Blocker™, Cook Incorporated, Bloomington, IN, USA; EZ-Blocker™, Rüsch, Teleflex Medical, Athlone, Irland; Ambu® VivaSight™ EBB, ETView Ltd., Amsterdam, The Netherlands) were available.

The SOP valid during the examination describes the insertion of a left-sided DLT as followed: After advancing the blue tip of the endobronchial lumen through the vocal cords, a 90° counterclockwise rotation of the DLT to the left (or in case of a right-sided DLT 90° clockwise to the right side) is done and the tube is advanced further into the trachea until a slight but springy resistance occurs. Diameter of the left main bronchus as depicted on CT scan was used to determine appropriate size of a DLT. The insertion depth of the tube at the corner of the mouth is estimated using the formula of Takita and coworkers (Depth = height in cm x 0.1 + 12.5 cm) [22].

During endotracheal intubation, the cuff should not contact the teeth of the patient to prevent cuff damage.

In a first step, correct DLT positioning is confirmed by bilateral auscultation after blocking the tracheal cuff and subsequently the endobronchial cuff, hearing breathing sounds over both lungs. After clamping and disconnection of the tracheal lumen, a left-sided DLT exhibits remaining breath sounds on the left thorax during ventilation. After declamping, reconnection and ventilation of the tracheal lumen, clamping and disconnection of the endobronchial lumen follows. Breath sounds of the right lung could be heard. With a right-sided DLT, procedure is the other way round.

The second step is mandatory bronchoscopic visualization of the blue endobronchial cuff through the tracheal lumen. The endotracheal cuff should be located approximately 5 mm below the carina. Via bronchoscope, upper and lower lobe bronchus should be visible through the endobronchial lumen of a left-sided DLT.

For any unexpected intraoperative ventilation problem, immediate bronchoscopy must rule out secondary dislocation or any other obstruction of the tube or of the airway by mucus or blood.

### Implementation of vDLT

As bronchoscopy is extensively aerosol-generating [8–11], methods to avoid aerosol release by means of airtight covers were implemented for bronchoscopy and all other airway management maneuvers [23–25]. In the second approach, we identified the Ambu® VivaSight™ vDLT as an alternative to left-sided cDLT in adults. The design of the vDLT is identical to that of left-sided cDLTs and it is available in 35, 37, 39 and 41 Fr. The built-in camera in the distal tracheal lumen outlet is connected to an external monitor, allowing continuous visual monitoring of tube placement and tube location.

Therefore, to avoid bronchoscopy procedures, sufficient amounts of VivaSight™ vDLTs were obtained, and the anesthesiology teams were trained in a proper use in the first part of April 2020.

The SOP was adapted during April 2020, and the vDLT was introduced as a preferred left-sided DLT in adults during pandemic. The mandatory bronchoscopy was replaced by the use of the vDLT.

Regardless this, anesthesiologists were free to choose left-sided cDLTs with bronchoscopic control as well as right-sided cDLT if indicated or bronchial blocker to enable one lung ventilation.

All devices for one lung ventilation were used according to SOPs that were digitally available at the integrated anesthesia workstation.

Pandemic situation required multiple and profound measures especially in the field of airway management, but also in perioperative patient care. According to government regulations, all patients were tested for SARS-CoV-2 using a PCR test upon admission to the hospital and before transfer to the operating room accompanied by specific anamnesis. SARS-CoV-2 positive patients were treated in a separate wing of the operating room. Furthermore, they received additional security measures regarding bronchoscopy and unprotected endotracheal tube (ETT) ports. Bronchoscopy was performed with an airtight shield (endoscopy camera cover) via a non-ventilated limb of the DLT, and unprotected ETT ports were closed with an appropriate high-efficiency particulate air (HEPA) respiration filter.

In March 2020, all medical staff of the department of anesthesiology were instructed in doffing and donning personal protective equipment. During the first pandemic months, mask airway for anesthesia and mask ventilation during anesthesia induction were mostly replaced by rapid sequence intubation procedures. Port disconnection and bronchoscopies should be always carried out inside a non-ventilated limb of the DLT. To achieve this, respiration was stopped and disconnected proximal of the HEPA filter unless PEEP was released with respiratory filter protection. The designated limb was clamped at the Y-piece and respiration was connected and started again as one lung ventilation. Now, the non-ventilated limb of the DLT could be disconnected and if applicable bronchoscopy could be performed via the non-ventilated limb. From April 2020, FFP2 masks were in permanent use by all anesthesiology staff during patient treatment. In case of aerosol generating procedures in SARS-CoV-2 positive patients, FFP3 masks were mandatory.

Data analysis included patient demographic data like age, height, weight, sex, as well as ASA-classification, primary disease, surgical procedure, clinical experience of the responsible anesthesiologist and the surgical procedure. Furthermore, the airway device used for one lung ventilation, problems with placement of the device and during one lung ventilation (e.g. malposition or insufficient lung collapse) as well as oxygenation during placement and one lung ventilation were recorded.

Data were anonymized for the statistical analysis. Descriptive and explorative statistical analysis was done using the statistic program Statistica^®^ 6.2 (StatSoft Europe GmbH, Hamburg, Germany). Categorial variables are expressed as absolute numbers and percentages and continuous variables are presented as medians and interquartile range. Differences between the vDLT and the cDLT group are analyzed with the Mann-Whitney U-test (continuous variables). Statistical significance was set at *p* < 0.05.

## Results

From April 1st, 2020 to December 31st, 2021, a total of 800 anesthesia records of adult patients with thoracic surgical procedures in the department of thoracic surgery were identified. In 451 cases, a DLT was used to facilitate one lung ventilation. Out of these cases 343 patients received left-sided vDLT, 100 patients received left-sided cDLT and 8 patients received right-sided cDLT. Those with right-sided cDLT were excluded from further analysis.

Despite a large group of users including resident physicians supervised by a senior consultant physician, implementation succeeded easily and the device was well accepted.

Demographic data of the patients with vDLTs and cDLTs are shown in Table 1. There were no significant differences between height, weight and body mass index between both groups. However, patients in the vDLT group were significantly older than patients in the cDLT group.

The lowest oxygen saturation between the application of the induction drugs and the intubation as well as during the phase of the one lung ventilation was recorded in the vDLT group and the cDLT group. There were no significant differences between these both groups.

**Table 1:** Demografic data: Age, weight, height, BMI and lowest oxygen saturation during induction and one lung ventilation represented as median and interquartile range; * p = < 0.05; Basic airway characteristics are presented as total number and percent

	vDLT	cDLT
Sex	male: 224 female: 119	male: 57 female: 43
Age (years)	64.0 [55.0–74.0]	60.0 [53.0-69.5] *
Height (cm)	172.0 [164.0-180.0]	174.0 [165.5–182.0]
Weight (kg)	77.0 [64.0–88.0]	78.0 [68.5–90.0]
Body mass index	25.8 [22.4–29.1]	25.65 [22.35–29.35]
ASA score	I: 11 (3.2%) II: 144 (42%) III: 178 (51.9%) IV: 10 (2.9%) V: 0 (0%)	I: 5 (5%) II: 36 (36%) III: 53 (53%) IV: 6 (6%) V: 0 (0%)
Mallampati score	I: 43 (12.5%) II: 92 (26.8%) III: 22 (6.4%) IV: 2 (0.6%) Not specified: 184 (53,6%)	I: 12 (12%) II: 24 (24%) III: 8 (8%) IV: 0 (0%) Not specified: 56 (56%)
Cormack/Lehane score	I: 236 (68.8%) II: 59 (17.2%) III: 9 (2.6%) IV: 1 (0.3%) Not specified: 38 (11.1%)	I: 83 (83%) II: 13 (13%) III: 1 (1%) IV: 0 (0%) Not specified: 3 (3%)
DLT size	35 Ch: 42 (12.2%) 37 Ch:129 (37.6%) 39: Ch:172 (50.1%)	35 Ch: 7 (7%) 37 Ch:48 (48%) 39: Ch:45 (45%)
lowest oxygen saturation during induction	99.0 [98.0–100.0]	99.0 [98.0–100.0]
lowest oxygen saturation during one lung ventilation	96.0 [94.0–98.0]	96.0 [93.0–98.0]

### Perioperative procedural times

Key performance indicators are shown in Table 2. Independent anesthesia times like Anesthesia Induction Time (K2) and Anesthesia Emergence Time (K3) were comparable between the vDLT and cDLT group.

The Incision-to-Closure Time (K8) was significantly longer in the vDLT group than in the cDLT group. This significant difference in the Incision-to-Closure Time was also transferred to other times that includes this timespan.

**Table 2:** Perioperative procedural times in minutes represented as median and interquartile range; * *p* = < 0.05; Key performance indicators: K11: Start Presence Anesthesia Nursing Staff (A4) to End Presence Anesthesia Nursing Staff (A10); K12: Start Presence Anesthesiologist (A5) to End Presence of Anesthesiologist (A12); K10: Anesthesia Ready (A7) to End Follow-up Surgical Measures (O11); K8: Incision (O8) to Closure (O9); K2: Start Anesthesia (A6) to Anesthesia Ready (A7); K3: End Follow-up Surgical Measures (O11) to End Anesthesia (A9). All times and coding according to The German Perioperative Procedural Time Glossary [26].

	vDLT	cDLT
Presence Anesthesia Nursing Staff K11	240.0 [199.0-295.5]	237.0 [185.0-284.5]
Presence Anesthesiologist K12	211.0 [179.0-261.0]	192.0 [161.0-246.0] *
Perioperative Time K10	141.0 [112.0-185.0]	125.0 [100.0-178.5] *
Incision-to-Closure Time	89.0 [61.0-131.0]	74.0 [48.5–123.0] *
Anesthesia Induction Time K2	37.0 [30.0–47.0]	36.0 [29.0–46.0]
Anesthesia emergence Time K3	9.0 [6.0–15.0]	9.0 [5.0-12.5]

Diagnoses and Surgery procedures are shown in Tables 3 and 4.

**Table 3:** Preoperative diagnosis presented as total number and percent

Diagnosis: n (%)		vDLT	cDLT	Overall
Neoplasm	Primary neoplasm of lung and bronchial tissue	92 (26.8%)	25 (25%)	117 (26.4%)
	Secondary neoplasm	65 (18.9%)	9 (9%)	74 (16.7%)
	Neoplasm of lung and bronchial tissue of unknown dignity	38 (11.1%)	9 (9%)	47 (10.6%)
	Neoplasm of Pleural tissue	3 (0.9%)	4 (4%)	7 (1.6%)
	Neoplasm of unknown dignity of the thoracic wall	1 (0.3%)	1 (1%)	2 (0.5%)
	Hodgkin lymphoma	0 (0%)	1 (1%)	1 (0.2%)
	Mediastinal neoplasm	10 (2.9%)	2 (2%)	12 (2.7%)
	**Total**:	**209 (60.9%)**	**51 (51%)**	**260 (58.7%)**
Trauma	Pneumothorax	17 (4.9%)	9 (9%)	26 (5.9%)
	Hemothorax	41 (11.9%)	17 (17%)	58 (13.1%)
	Rib fracture	3 (0.9%)	2 (2%)	5 (1.1%)
	Traumatic paresis of the diaphragm	1 (0.3%)	0 (0%)	1 (0.2%)
	**Total**:	**62 (18.1%)**	**28 (28%)**	**90 (20.3%)**
Other	Complication of a medical procedure: Stomach injury	0 (0%)	1 (1%)	1 (0.2%)
	Emphysema with bullae	4 (1.2%)	1 (1%)	5 (1.2%)
	Hernia of the chest wall	1 (0.3%)	0 (0%)	1 (0.2%)
	Thymoma	3 (0.9%)	0 (0%)	3 (0.7%)
	**Total**:	**8 (2.4%)**	**2 (2%)**	**10 (2.3%)**
Infectious diseases	Pleural empyema	21 (6.1%)	1 (1%)	22 (5.0%)
	Pleural effusion	30 (8.7%)	11 (11%)	41 (9.3%)
	Other pleural diseases	13 (3.8%)	3 (3%)	16 (3.6%)
	Echinococcosis of the lung	0 (0%)	1 (1%)	1 (0.2%)
	Aspergillosis of the lung	0 (0%)	1 (1%)	1 (0.2%)
	Lung abscess	0 (0%)	1 (1%)	1 (0.2%)
	Mediastinal inflammation	0 (0%)	1 (1%)	1 (0.2%)
	**Total**:	**64 (18.6%)**	**19 (19%)**	**83 (18.7%)**

Overt differences between vDLT and cDLT could be seen in neoplasm surgery (60.9% versus 51%) and surgery for pneumo- and hemothorax and trauma (18.1% versus 28%), depicting a possible discriminative selection of the devices. Incidence of surgery for infectious diseases were comparable between the vDLT group and the cDLT group (18.6% versus 19%).

**Table 4:** Surgical procedures presented as total number and percent

Surgical procedures: *n* (%)	vDLT	cDLT	Overall
Wedge resection	85 (24.8%)	20 (20%)	105 (23.7%)
Segment resection/ Bisegment resection	12 (3.5%)	6 (6%)	18 (4.1%)
Lobectomy/ Bilobectomy	46 (13.4%)	16 (16%)	62 (14.0%)
Pneumonectomy	1 (0.3%)	0 (0%)	1 (0.2%)
Other resection of the lung and bronchi	44 (12.8%)	7 (7%)	51 (11.5%)
Explorative thoracotomy	0 (0%)	4 (4%)	4 (0.9%)
Thoracoscopy/ Mediastinoscopy	28 (8.2%)	12 (12%)	40 (9.0%)
Hematoma evacuation via thoracotomy or video- assisted- thoracoscopy (VATS)	37 (10.8%)	17 (17%)	54 (12.2%)
Pleurectomy/ Pleural decortication	43 (12.5%)	10 (10%)	53 (12.0%)
Thymectomy	3 (0.9%)	0 (0%)	3 (0.7%)
Other surgical procedures of chest wall, pleura, mediastinum and diaphragm	44 (12.8%)	8 (8%)	52 (11.7%)

In the vDLT group 323 of the surgical procedures were planned, and 20 (5.8%) were emergencies. Of these emergencies, 9 cases were classified by the surgeon with priority level 4 (surgery in between 24 h), 3 cases were classified with priority level 3 (surgery in between 6 h), 6 cases were classified with priority level 2 (surgery in between 2 h) and 2 cases were classified with priority level 1 (immediate surgery).

In the left-sided cDLT group 95 of the surgical procedures were elective and 5 (5%) were emergencies.

The diagnoses of the 25 emergency patients are shown in Table 5.

**Table 5:** Diagnoses of priority classified emergencies presented as total number

Prioritity level	vDLT	cDLT
4 (< 24 h)	pleural effusion: 3 pleural empyema: 4 hemothorax: 1 pneumothorax: 1	-
3 (< 6 h)	pleural effusion: 2 pleural empyema: 1	lung abscess:1 pleural empyema: 1
2 (< 2 h)	pleural empyema: 2 hemothorax: 4	hemothorax: 2
1 (immediately)	hemothorax: 2	traumatic injury of the chest wall: 1

While cDLTs always required a bronchoscopic position check, in the vDLT group only 50 (14.6%) of the 343 patients required an additional bronchoscopy, which was in 38 cases (11%) because of anesthesiological requirement (reasons for bronchoscopy arising from the anesthesiological management of one lung ventilation like position control of the DLT). A non-anesthesiological requirement (reasons for bronchoscopy arising from the surgical or other requests; for example, control of the surgical result) was present in 12 cases (3.6%) in the vDLT and in 1 case (1%) in the cDLT group.

The reasons for additional bronchoscopy are listed in Table 6.

Notably, documented numbers of additional bronchoscopy in the cDLT group might be false low, as it had to be documented anyway.

**Table 6:** Reasons for additional bronchoscopy presented as total number and percent. *non-anesthesiological requirement for bronchoscopy

Indication for bronchoscopy	vDLT	cDLT
DLT correction during intubation	3 (0.9%)	0 (0%)
DLT control after intubation	7 (2%)	Routine control
DLT control after patient positioning	6 (1.7%)	Routine control
Airway obstruction with bronchial exudate	2 (0.6%)	2 (2%)
Intraoperative dislocation of the DLT	4 (1.2%)	2 (2%)
Problems to establish ventilation or ventilation problems during lung isolation	7 (2%)	3 (3%)
Insertion of an EZ-Blocker after switching rules	2 (0.6%)	1 (1%)
Restricted camera view due to fluids	7 (2%)	-
Control of surgical result*	5 (1.5%)	1 (1%)
Bronchial lavage for microbiologic specimen*	3 (0.9%)	0 (0%)
Bronchial lavage after surgery*	3 (0.9%)	0 (0%)
Bronchoscopic detection of broncho-pleural fistula*	1 (0.3%)	0 (0%)

### Difficult endotracheal intubation

In 10 cases (2.9%) of the vDLT group an unexpected difficult airway occurred. The initial placement of the vDLT required additional airway devices as Cook® airway exchange catheters, Eschmann’s rods, videolaryngoscopes and bronchoscopes. However, the initial chosen vDLT could be placed with the aid of the above-described airway devices.

An unexpected difficult airway was found in 5 cases (5%) of the cDLT group. The initial chosen cDLT could be placed with the aid of additional airway devices as videolaryngoscopes, Cook® airway exchange catheters and Eschmann´s rods.

### Change of device and lung isolation procedure

In five cases (1.45%) of the vDLT group the initial chosen vDLT had to be changed, because of cuff leakage or impossibility to place the left-sided vDLT in the left main bronchus. In three of this five cases, the tracheal cuff itself leaked immediately after the intubation, which were all difficult with a Cormack Lehane III view. Alternative management succeeded via single-lumen tube and a bronchial blocker in two of the latter cases. The third case could be solved downsizing to a 35 Fr vDLT. In the other two of this five cases a correct left main bronchus placement failed despite bronchoscopic support. Intubation and one lung ventilation were performed in both cases with a single-lumen tube with an EZ-Blocker™.

In one case (1%) of the cDLT group a correct placement of the endobronchial branch of the DLT in the left main bronchus failed and one lung ventilation was performed with a single-lumen tube and an EZ-Blocker™.

In another five cases (1.45%) of the vDLT group the initial chosen vDLT mismatched with the patient’s airway anatomy and a smaller vDLT was successful placed.

In one case (1%) of the cDLT group the initial chosen cDLT was too large. This problem was solved by a smaller cDLT, which could be placed without any problems.

### First pass success

In summary, in the vDLT group first pass success was 94.2% with initially correct placement of the initially selected vDLT size. No immediate success was found in 5.8% (20 out of 343 applications: 10 placements of the vDLT using other airway devices and five procedure changes as well as five smaller tubes).

In the cDLT group first pass success was 93%. No immediate success occurred in 7% (seven out of 100 applications: five placements of the cDLT using other airway devices and one procedure change as well as the needfulness of a smaller cDLT in one case).

### Line-of-sight obstruction

The irrigation port directed toward the camera for flushing the camera of mucus with air or saline worked properly. Persisting blurring of the vDLT camera with fluids and mucus – despite flushing with air or saline solution occurred in 7 cases (2%), requiring confirming additional bronchoscopy.

## Discussion

DLTs are representing the preferred device for intraoperative one lung ventilation in thoracic surgery. Initially insufficient positioning occurs in 4.2–39% of cases. Auscultation alone is not sufficient to check the correct position and a bronchoscopic confirmation is required both after intubation and following transfer into side position [1–3, 17, 27]. Seo and colleagues identified choosing too small sizes of DLTs as a predictor for incorrect positioning of a left-sided DLT into the right main bronchus, in addition to a short and tiny stature and female gender [27]. Due to a potentially limited lung function, fast and correct placement of the DLT has top priority in thoracic surgery patients [18]. Incorrect positioning therefore bears an immediate and high risk of hypoxia [28, 29]. In cDLTs, bronchoscopy is required to confirm and correct position of the tube. Both deterioration in oxygenation during repositioning and aerosol generation with subsequent contamination of staff are important risks [12]. Numerous medical societies and clinical institutions recommendations and checklists deal with staff exposure avoidance by minimizing aerosolization and reducing the time of an unprotected airway [8–11, 30]. Practical implementation resulted in various protective aids as aerosol boxes, protective barriers like covers for bronchoscopes in order to minimize the spread of aerosols [23–25].

In order to reduce routine fiberoptic bronchoscopic exam to ensure optimal position the Ambu® VivaSight™ vDLT represents an alternative to the use of a cDLT [19].

During the retrospective period, 77.4% of all DLT devices used were vDLTs. A possibly overt bias occurred regarding the type of surgery. In neoplasm surgery, vDLT was used more often than in small interventions like trauma surgery with presumably younger patients. This might explain the significantly larger proportion of elderly patients and longer perioperative times in the vDLT group. Due to the retrospective study approach, no causal explanation of this distribution is possible. One can speculate, that anesthesiologists might considered the relatively new vDLT procedure as more time-consuming and not worth for minor intervention.

### Need of additional fiberoptic bronchoscopy in vDLT

Proportion of bronchoscopy with the use of the vDLT was less (14,6%) when compared with the cDLT (100% use). As the overall rate of additional bronchoscopy was 14.6%, only 11% of the cases were related to anesthesiological requirements - both a result comparable to findings in literature, where a need for fiberoptic bronchoscopy (FOB) with possible aerosol-generation is described using vDLTs with an incidence between 6.6 and 28% in different studies with 30 to 80 study participants [14–17].

### First pass success tube placement, camera view and malpositions

As depicted above, in most studies of the vDLT, only relatively small group sizes were examined [1, 16, 17]. Massot and colleagues planned to evaluate the rate of correct positioning of the vDLT in a prospective observational study in 170 patients. After 84 patients being included, the study was terminated because one vDLT melted before insertion. Out of 77 evaluable cases, the vDLT could be positioned correctly in 99% (76/77). In 53% of these cases intraoperative malpositions occured. The authors concluded that a continuous visualization of the carina is a major improvement for patient care facilitating immediate recognition and reposition of intraoperative displacement [19].

Rapchuk and coworkers evaluated the vDLT over a study period of six months after introduction in a single institution. In 100 operations, a vDLT was used in 72 cases, a cDLT was used in 27 cases and a bronchial blocker was used for one lung ventilation in one case. The vDLT could initially be placed correctly in 85%. Camera view was good in 75% of the cases but required flushing with air or saline in 39% of all cases [21]. Onifade and Coworker described that the camera of the vDLT was laid with mucus in 48% of cases and was easily cleaned in 42% [17].

Only in 7 cases (2%) of our 343 vDLT applications these flushing procedures could not reestablish a sufficient camera view and a confirming bronchoscopy was required to clean the camera of the vDLT. In line with previous findings, we found predominatly undisturbed and continuous view of the carina.

The latter study found intraoperative dislocations due to surgical manipulation, hyperextension of the head and neck, or an over-blocked bronchial cuff occurred in 21 of 72 cases (29%). In 19 of the cases, repositioning was successful using the built-in camera of the tube and only in 2 cases (2.8% of all cases) bronchoscopy was required [21]. The authors conclude, that the vDLT allows a fast identification and correction of intraoperative airway problems like malposition of the DLT during one lung ventilation. In our study documented repositioning rate during one lung ventilation was 1.2% (4 cases) in the vDLT group and 2% (2 cases) in the cDLT group. Use of additional bronchoscopy because of problems to establish ventilation or ventilation problems during lung isolation occurred in 7 cases (2%) in the vDLT group and 3 cases (3%) in the cDLT group.

Schuepbach and coworkers enrolled 40 patients 1:1 to a cDLT group and a vDLT group with respect to time to intubation and insertion success without bronchoscopy, frequency of tube displacements and ease of insertion. 100% of the vDLTs were correctly inserted while in the cDLT group only 85% of the tube were initially correct inserted. They found a significant shorter time to intubation in the vDLT group as well as Onifade and colleagues [17, 18]. Levy-Faber and colleagues reported 32 out of 35 intubations at first attempt in a vDLT group versus 33 out of 36 in a cDLT group. However, intubation time was significantly decreased in the vDLT group with a median of 51 s when compared to the cDLT group with a median of 264 s [31].

In our study the vDLT enables with 94.2% a very high, but no total first pass success. In 10 cases (2.9%) the initial placement of the selected vDLT required additional airway tools as videolaryngoscopes or airway exchange catheters due to a difficult airway situation. In five cases (1.45%) the initial chosen vDLT mismatched with the patient’s airway anatomy and a smaller vDLT was successfully placed. In additional five cases (1.45%) the initial chosen vDLT had to be changed, because of cuff leakage or impossibility of placement in the left main bronchus. In three of this five cases, the tracheal cuff itself leaked immediately after the intubation, which were all difficult with a Cormack Lehane III view. Most likely, the cuff encountered the patient’s teeth. Time to intubation was not part of the analyzed routine data. The anesthesia induction time (induction and intubation, insertion of arterial and central venous line) are not different between the vDLT group and the cDLT group in our collective. From the clinicians point of view, process times should be shorter with a vDLT and without additional bronchoscopy. As routine bronchoscopy with cDLT is usually performed parallel to surgical positioning and skin disinfection, there might be no measurable effect of this advantage. Koopman and coworker found in a prospective multicenter study with 151 patients a correct placement of the vDLT 37 Fr in 148 patients. Placement of the vDLT was difficult in 19%. Reasons were poor identification of the carina or purely subglottic resistance during placement [20]. However, not all vDLT sizes were available at the time of Koopman’s study [20]. Therefore, it might be essential that a full set of vDLT sizes is locally available and could be purchased.

### Limitations

Due to the retrospective character the present study might have some potential limitations. Like any retrospective analysis, there is a high dependency on the quality and the completeness of the documentation. These requirements were able to be met by the long-established documentation in the electronic anesthesia data management system NarkoData®. This system has several mandatory entry fields so that a high degree of reliability in terms of completeness of the documentation was fulfilled. Because of routine bronchoscopic control of the cDLT after intubation and after patient positioning as well as the continuous view to the carina with the vDLT only those intraoperative malpositions were documented, that required additional bronchoscopy. The retrospective nature of this study implies that the clinical circumstances and conditions were not uniform for every case. However, this is precisely what accounts for the advantage of the present analysis, that it was used in a broad clinical area and not under narrowly limited study conditions. Furthermore, the vDLT was recommended as the preferred left-sided DLT to enable one lung ventilation in adults during SARS-CoV-2 pandemic but regardless this recommendation anesthesiologists were free to choose left-sided cDLTs with bronchoscopic control as well. Depending on own previous experience of the training assistants and specialists or the experience of the supervising senior physician with both devices, cDLT or vDLT, a bias regarding the selection of the used DLT cannot ruled out. Ultimately, there was a 77.4% rate of compliance with the institutional recommendation to prefer vDLT for generation of one lung ventilation during SARS-CoV-2 pandemic.

## Conclusions

The Ambu® VivaSight™ vDLT is an efficient, easy-to-use and safe airway device for the generation of one lung ventilation in patients undergoing thoracic surgery. Using the vDLT can reduce the need for aerosol-generating bronchoscopic interventions by 85.4%. This increases employee’s safety during SARS-CoV-2 pandemic as well as in exposition to other pathogens. The vDLT-staff-implementation was achieved easily. Continuous video view to the carina enabling position monitoring might be beneficial for both employee’s and patient’s safety.

## Data Availability

Please contact corresponding author for data requests.

## Abbreviations

DLT: Double-lumen tube
cDLT: conventional double-lumen tube
vDLT: Video double-lumen tube
Fr: French
SOP: standard operating procedure
VATS: video- assisted- thoracoscopy
FOB: fiberoptic bronchoscopy
